# Diagnostic tools of caprine and ovine anaplasmosis: a direct comparative study

**DOI:** 10.1186/s12917-018-1489-x

**Published:** 2018-05-22

**Authors:** I. I. Shabana, N. M. Alhadlag, H. Zaraket

**Affiliations:** 1 Biology Department, Faculty of Science, Taibah University, Al- madinah Al-munawarah, Saudi Arabia; 2 Faculty of Veterinary Medicine, Department of Bacteriology, Immunology and Mycology, Suez Canal University, Ismailia, Egypt; 3 Department of Experimental Pathology, Immunology & Microbiology, Faculty of Medicine, American University of Beirut, Beirut, Lebanon; 4 Center for Infectious Diseases Research, Faculty of Medicine, American University of Beirut, Beirut, Lebanon

## Abstract

**Background:**

The diagnosis of anaplasmosis is rather conflicting with other haemoprotozoans. Hence, the study aimed to compare and evaluate the efficiency of competitive ELISA (cELISA), indirect fluorescence antibody (IFA), and Polymerase chain reaction (PCR) for precise diagnosis of *Anaplasma* spp. and to assess their concordance with microscopic examination (ME).

**Results:**

A total of 312 blood samples (189 sheep and 123 goats) were examined for *Anaplasma* infection during a 1 year period. Giemsa-stained blood smears were examined under the microscope. IFA and cELISA were used for the detection of *Anaplasma* spp. antibodies. PCR was used as a standard of truth and for the identification of *Anaplasma* species. Using cELISA assay, 47.4% (148) were positive (93 sheep and 55 goats) with a sensitivity and specificity of 91.9, and 86.9%, respectively. Using IFA, it was found that 57.4% (179)were positive (113 sheep and 66 goats) with a sensitivity and specificity of 100, and 93.3%, respectively. PCR assay identified *A. ovis* in 49 (25.3%) sheep and 30 (15.5%) goats, and *A. phagocytophilum*in 74 (38.1%) sheep and 41 (20.8%) goats.

**Conclusions:**

High sensitivity and specificity values of IFA and ELISA tests compared to microscopic examination strongly support their utility in the diagnosis of *Anaplasma* infection. PCR was a more specific diagnostic tool that allows to discriminate between *Anaplasma* subspecies*,* which makes it the method of choice for anaplasmosis diagnosis.

## Background

*Anaplasma* are obligate intracellular, Gram-negative, tick-borne rickettsial bacteria that are important animal and human pathogens. Anaplasmosis constitutes a burden to livestock health and production in tropical and sub-tropical regions. It results in great economic losses due to decreased production, mortality, and lowered work efficiency of affected animals [7, 26].There are six recognized *Anaplasma* species: *A. ovis, A. marginale, A. centrale, A. platys, A. bovis,* and *A. phagocytophilum* [48]. *A. ovis* primarily infects sheep and goats, causing ovine and caprine anaplasmosis, respectively [24]. *A. ovis* infection is generally a subclinical or mild condition, while severe infection may involve anemia, abortion, and mortality [43].

*A. phagocytophilum* infects humans and animals and is thought to be zoonotic [38, 49]. The disease is known as tick-borne fever (TBF) in human and ruminants granulocytic anaplasmosis (HGA) in human. Tick-borne fever is characterized by high fever, which may last for one to 2 weeks, followed by a severe neutropenia, which makes the animals susceptible to secondary infections [39, 40]. Ruminants granulocytic anaplasmosis is characterized by fever, myalgia, chills, depression, and headache [47]. Anaplasmosis became a notifiable disease in 1999 [11, 20].

Anaplasmosis diagnosis is usually based on the microscopic examination (ME) of Giemsa-stained blood smears, serological, and molecular diagnostic procedures. Microscopic diagnosis may be difficult in carrier animals, thus various serological techniques have been used for the detection of *Anaplasma*-specific antibodies such as indirect immunofluorescence antibody (IFA), enzyme-linked immunosorbent assay (ELISA), and complement fixation tests (CF) [9, 23].The competitive ELISA (cELISA) is depending on the use of a monoclonal antibody (Mab) ANAF16C1 that recognizes the conserved (MSP-5) antigen of different *Anaplasma* spp. [28, 46]. cELISA test has high sensitivity and specificity for detection of *Anaplasma* antibodies [27].

The “Gold standard” method for the diagnosis of *Anaplasma* spp. relies on the combination of the microscopic examination and cELISA [22].The indirect immunofluorescence antibody test is widely used for the diagnosis of blood protozoon and Rickettsia [25].The IFA test is commonly used in epidemiological studies because of its low costs [14, 25].

Molecular identification methods such as Polymerase chain reaction (PCR) have several advantages compared to the traditional serologic and blood smear tests [21]. PCR is the most sensitive and reliable diagnostic tool that allows discriminating between *Anaplasma* subspecies [44]. In addition, PCR can detect the coinfections with multiple *Anaplamsa* subspecies [5].

The aim of the study was to assess the sensitivity and specificity of the different diagnostic tools used for detecting anaplasmosis in sheep and goats.

## Methods

### Study design

This was a prospective study conducted according tothe Standards for Reporting Diagnosis Accuracy Studies (STARD) [13].

### Animals

**Fig. 1:**
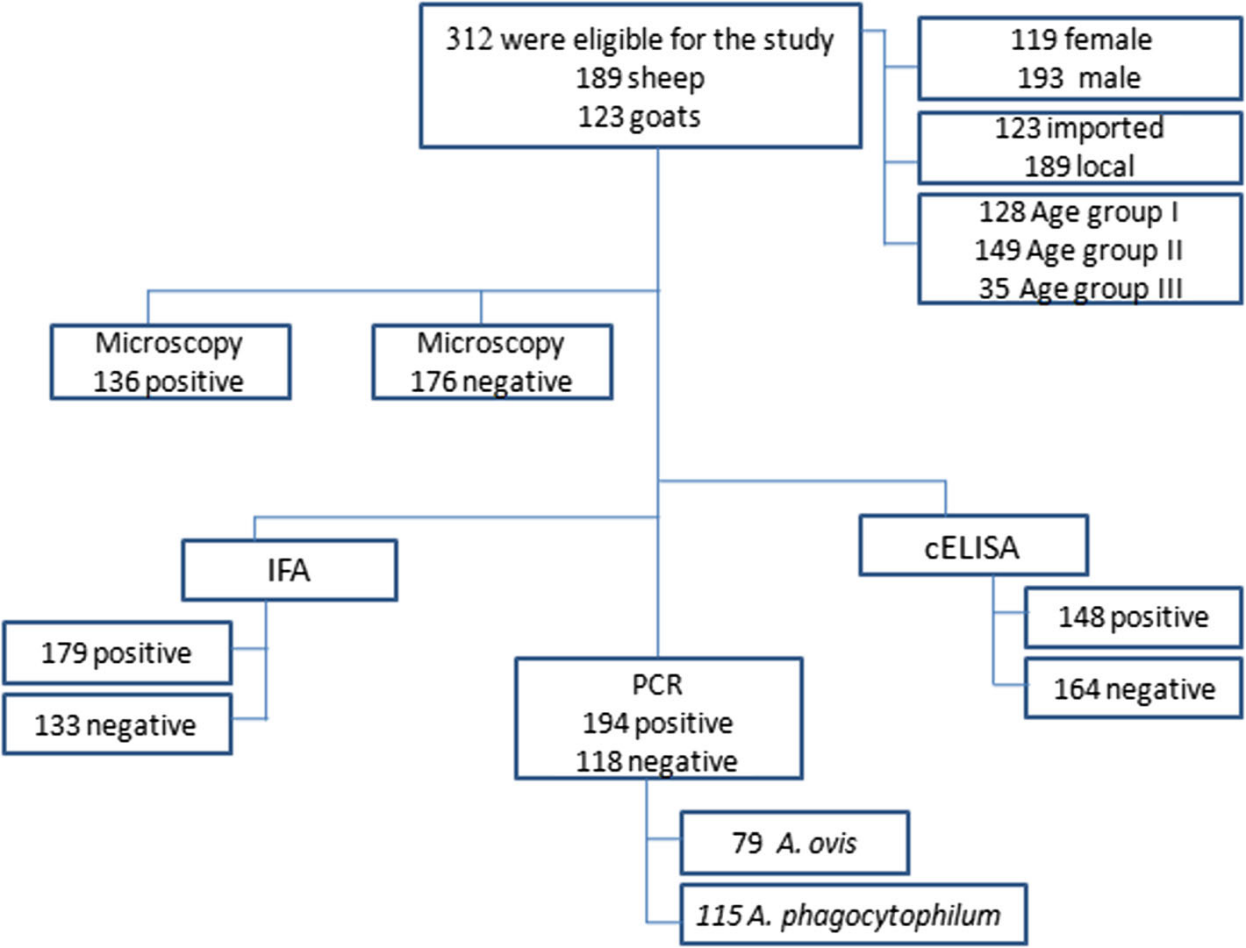
Study flow chart

Blood samples were collected from sheep and goats from the local abattoir and different farms in Medina, Kingdom of Saudi Arabia (KSA). Eligibility criteria were suspicion of anaplasmosis among sheep and goats in Medina, which has a subtropical climate and borders on a fully tropical climate. The animals were surveyed for anaplasmosis from September 2011 to November 2012 (Fig. 1). Microscopic examination of blood smears was basically used as a reference diagnosis of anaplasmosis. cELISA and IFA are the most commonly used serological methods to detect antibodies against *Anaplasma*. PCR is the most reliable diagnosis of *Anaplasma* infection.

### Blood samples collection

A total of 312 blood samples were collected from 189 sheep and 123 goats of different age groups (2 months to 6 years) and sources (local and imported). The imported animals were either from Sudan, Pakistan, Australia, and Somalia. Venous blood samples were collected from each individual animal in two 4 ml vacutainer tubes (BD vacutainer, BD-Plymouth, UK), one containing EDTA and one additive-free. To separate sera, the additive-free blood was allowed to clot for about 15–30 min at room temperature. The tubes then centrifuged at 1000–2000 rpm for 10 min and serum was collected. The serum specimens were stored at − 20 °C for further use.

### Microscopic examination

Thin blood smears were prepared for microscopic examination accordingly the standard protocol [7].The slides were allowed to air-dry before being fixed with absolute methanol. Fixed smears were stained with 10% Giemsa (Cresent diagnostic, KSA) and examined by using compound microscope under oil immersion lens. About 25 fields were examined from each slide for the presence of *Anaplasma* and the number of infected erythrocytes. *Anaplasma* was identified on the basis of its morphology [8].

### Competitive ELISA (cELISA) assay

Sera were screened for the presence of *Anaplasma* antibodies by using the VMRD cELISA kit (VMRD Inc., Pullman, WA, USA), according to the manufacturer instructions. The optical density (OD) was measured at 650 nm with an ELISA microplate reader MR-960 (perlong Medical Equipment Co., Ltd., China). The results were calculated according to the formula: 100 [1-(Sample OD ÷ Negative Control OD)]. The positive sample OD must be > 30%.

### Immunofluorescence (IFA)assay

Sera were screened for *Anaplasma* immunoglobulin G (IgG) by a semi-quantitative indirect IFA commercial kit (Fuller, USA), according to the manufacturer instructions. Briefly, sera samples were diluted in phosphate-buffer saline (PBS) and 25 μl were transferred to the slide wells. The slides were incubated at 35 °C for 30 min then washed with PBS followed by distilled water to remove the unreacted antibodies. Twenty five μl anti-ovine conjugate with DyLight 488 dye (Fuller, USA) were added and incubated then removed by washing as previously described. The slide was examined by standard fluorescence microscopy (Olympus BX50, Japan) at 400X magnification, the positive reaction appears as green fluorescent small cocci with a red background.

### DNA extraction and PCR

DNA extraction was carried out using the G-spinTM Total DNA Extraction Kit (iNtRON Biotechnology, Korea) according to the instructions of the manufacturer. PCR was performed to detect both *Anaplasma phagocytophilum*, *Anaplasma ovis* using BioinGentech Veterinary PCR Kits (Concepcion, Chile) according to the instructions of the manufacturer. The cycling conditions were initial denaturation at 94 °C for 2 min, 35 cycles (94 °C 30 s, 57 °C30 s, 72 °C 30 sec) and a final extension at 72 °C for 5 min.

### Statistical analysis

Statistical analysis was performed using the Statistical Package for the Social Sciences version 20.0 (SPSS Inc. Chicago, USA). Eta-square and Chi-square were applied to compare the percentage of positive samples by each method. The differences were considered statistically significant when *P*-value is< 0.05.

## Results

### The examined animals

A total of 189 (60.6%) paired samples were collected from sheep and 123 (39.4%) were obtained from goats. As shown in (Table 1), the animals were classified into three age groups: group I included samples collected from animals aged2 months to 2 years (128; 41.5%), group II included animals > 2 -4 years (149; 47%), and group III included animals > 4 – 6 years (35; 12%). According to the country of origin,189 (60.6%) of the sampled animals were locally bred and included 107 (56.6%) samples collected from sheep and 82 (43.4%) samples of goats, and 123 (39.4%) animals were imported including 82 (66.7%) sheep and 41(33.3%) goats.

**Table 1:** Basic demographics of the sampled animals

Categories		*N* (%)
Gender	Female	119 (38.1%)
	Male	193 (61.9%)
Host	Sheep	189 (60.6%)
	Goat	123 (39.4%)
Source	Imported	123 (39.4%)
	Local	189 (60.6%)
Age	Age group I (2 m-2y)	128 (41.5%)
	Age group II (2y-4y)	149 (47%)
	Age group III (4y-6y)	35 (12%)

### Microscopic examination

**Fig. 2:**
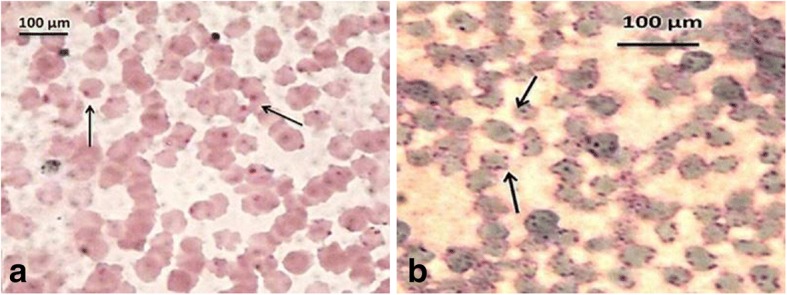
**a**, **b** Anaplasma species under microscope appears as small and roughly spherical intraerythrocytic parasite measuring about 0.2 to 0.5 μm

A total of 312 blood smears were examined for the presence of intra-erythrocytic inclusions using Giemsa stain. *Anaplasma* spp. appeared as small spherical deep purple intraerythrocytic inclusions, measuring about 0.2 to 0.5 μm (Fig. 2a, b). Among the collected specimens, 130 were found positive with an overall prevalence of 43.6%. Among the 130 positive animals, 86 (45.5%)were sheep and 50 (40.7%) were goat (Table 2). The difference in Anapalsma prevalence in sheep and goats was not significant (*P* > 0.05).The highest infection rate of *Anaplasma* spp. was among animals aged 2 m–2y, in sheep (47/86; 54.6%)and goats (22/50; 44%) with a significant correlation (*P* < 0.05). The infection rate was higher albeit not significantly among the imported animals, 64.6% in sheep and 82.9% in goats compared to locally bred animals. Male animals had a higher and significant (*P* < 0.05) infection rate in sheep (66/86; 76.7%) and goats (38/50; 76%) compared to females.

**Table 2:** Microscopic examination

Host	Demographic factor		Positive animals percentage
Sheep (*n* = 189)	Gender	Male (128)	66 (34.9%)^a^
		Female (61)	20 (10.6%)
	Age	Group I (84)	47 (24.9%)^a^
		Group II (89)	36 (19.0%)
		Group III (16)	3 (1.6%)
	Source	imported (82)	53 (28.1%)
		Local (107)	33 (17.5%)
Goat (*n* = 123)	Gender	Male (65)	38 (30.9%)^a^
		Female (58)	12 (9.8%)
	Age	Group I (44)	22 (17.9%)^a^
		Group II (60)	24 (19.5%)
		Group III (19)	4 (3.3%)
	Source	Imported (41)	34 (27.6%)
		Local (82)	16 ( 13.0%)

Age group I (2 months to 2 years), group II (> 2 -4 years), group III (> 4 - 6 years)

^a^Significant variation

### Competitive inhibition ELISA (cELISA) assay

The overall prevalence of *Anaplasma* spp. using cELISA was 47.4% (*n* = 148), including 93 (49.2%) specimens from sheep and 55 (44.7%) from goats. In sheep, the infection rates were higher among males (37%), animals of the age group I (61.1%). and imported animals (71.1%). While in goats, the prevalence was 60% among males, 59.1% among age group I animals, and 87.8% in the imported animals (Table 3). Comparing the data obtained from microscopic examination with cELISA, it was observed that cELISA has high sensitivity (91.9%) and specificity (86.9%) than microscopic examination. The Gold standard of truth for the diagnosis of *Anaplasma* spp. relies on the combination of the microscopic examination and cELISA.

**Table 3:** Seroprevalence of anaplasma species among sheep and goats

Host	Serological assay	Gender		**Age			Source	
		Female	Male	Group I	Group II	Group III	Imported	Local
Sheep	cELISA	70 (37.0%)	23 (12.2%)	52 (61.1%)	39 (43.8%)	2 (12.5%)	59 (71.1%)	34 (31.7%)
	IFA	82 (43.4%)	31 (16.4%)	60 (31.7%)	45 (23.8%)	8 (4.2%)	69 (36.5%)	44 (23.3%)
Goat	cELISA	39 (60.0%)*	16 (27.6%)	26 (59.1%)	25 (41.7%)	4 (21.1%)	36 (87.8%)	19 (23.1%)
	IFA	45 (36.6%)*	21 (27.6%)	30 (24.4%)	29 (23.6%)	7 (5.7%)	43 (34.9%)	23 (18.7%)

* statistically significant (*P*-value is < 0.05)

**statistically highly significant (*P*-value is < 0.01)

### Immunofluorescence (IFA)assay

**Fig. 3:**
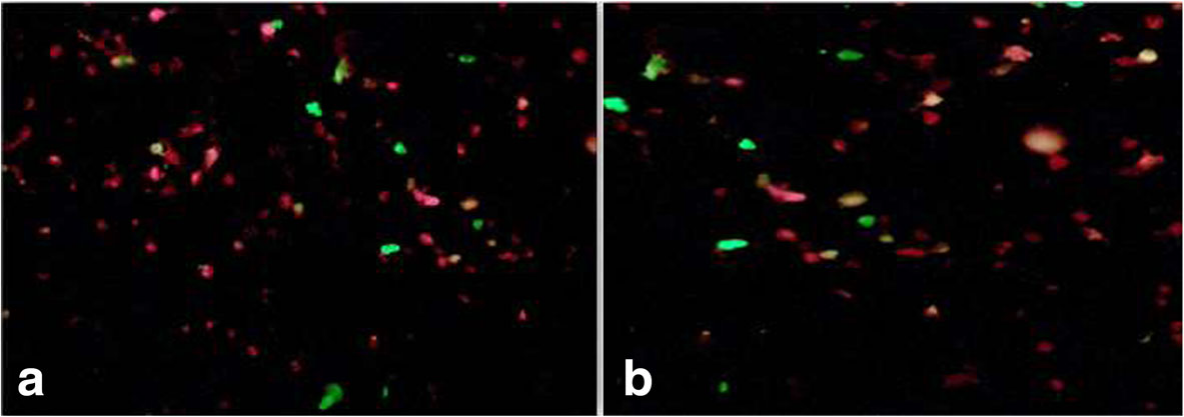
**a**, **b** Anaplasma species under fluorescent microscope appears green fluorescent small cocci with a red back ground

The presence of the intraerythrocytic *Anaplasma* spp. is indicated by greenish yellow or yellowish fluorescence (Fig. 3a, b). The overall prevalence of *Anaplasma* spp. using IFA was 57.4% (179), of which113 (59.8%) were sheep and 66 (53.7%) were goats (Table 3). In sheep, the infection rate was higher among males (43.4%), animals of the age group I (31.7%), and imported animals (36.5%). While in goats, the prevalence was 36.6% among males, 24.4% among age group I animals, and 34.9% in imported animals. The agreement between positive and negative results in cELISA and IFA were 82.7% (148/179). The remainder 17.3% (31 samples) was represented by samples, which were positive in IFA and negative in cELISA. No samples were found to be negative in IFA but positive in cELISA. The Gold Standard for the detection *of Anaplasma* spp. is the concordance of both cELISA and microscopic examination results. The sensitivity and specificity of IFA were 100 and 93.3% respectively, compared with the gold standard (Table 4).

**Table 4:** Sensitivity and specificity of IFA as compared with gold standard (ELISA and ME) of anaplasma spp.

	Gold Standard			
		Positive	Negative	Total
IFA test	positive	125	11	136
	Negative	0	153	153
	Total*	125	164	289

* statistically significant (*P*-value is < 0.05)

**statistically highly significant (*P*-value is < 0.01)

### PCR analysis

**Fig. 4:**
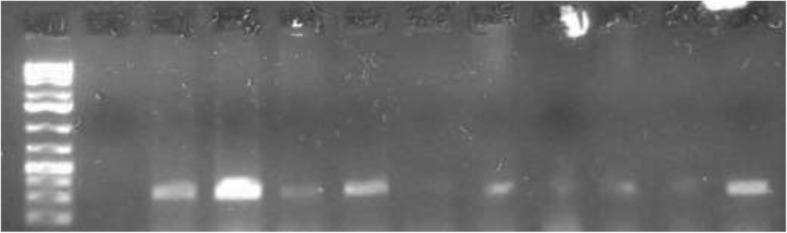
Agarose gel electrophoreses of PCR products of *Anaplasma ovis* amplified from DNA purified from sheep and goats blood samples. Right arrowhead indicates position of the 271 bp PCR product

**Fig. 5:**
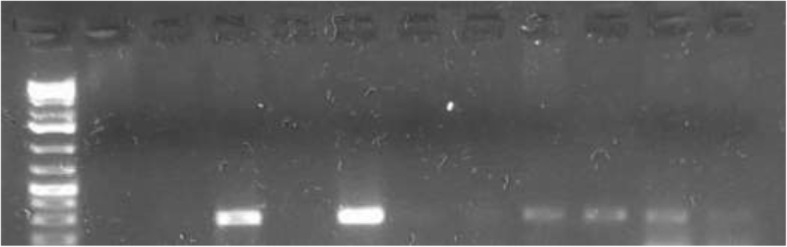
Agarose gel electrophoreses of PCR products of *Anaplasma phagocytophilum* amplified from DNA purified from sheep and goats blood samples. Right arrowhead indicates position of the 236 bp PCR product

Molecular identification of *Anaplasma* spp. revealed two distinct species: *Anaplasma ovis* (Fig. 4) and *Anaplasma phagocytophilum* (Fig. 5). *Anaplasma ovis* was detected in 25.3% (25.9% of sheep and 24.4% of goats) of the sampled animals, while *Anaplasma phagocytophilum* was detected in 36.9% (39.2% of sheep and 33.3% of goats). Mixed infections with the two species were detected in18.9% of the animals (20.6% of sheep and 16.3% of goats).The sensitivity and specificity of PCR assay was 73.2 and 89% respectively when compared with microscopic examination (Table 5).

**Table 5:** Molecular identification

	Anaplasma spp.	Gender		**Age			Source	
		Male	Female	Group I	Group II	Group III	Imported	Local
Sheep	*A. ovis*	35 (18.5%)	14 (7.4%)	26 (13.8%)	23 (12.2%)	0	30 (15.9%)	19 (10.0%)
	*A. phagocytophylum*	50 (26.5%)	24 (12.7%)	37 (19.6%)	31 (16.4%)	6 (4.8%)	38 (20.1%)	36 (19.0%)
Goat	*A. ovis*	*19 (60.0%)	11 (27.6%)	13 (15.4%)	13 (15.4%)	4 (3.3%)	16 (13.0%)	14 (11.4%)
	*A. phagocytophylum*	20 (16.3%)	21 (17.0%)	23 (18.7%)	16 (13.0%)	2 (1.6%)	15 (12.2%)	26 (21.1%)

* statistically significant (*P*-value is < 0.05)

**statistically highly significant (*P*-value is < 0.01)

## Discussion

Anaplasmosis frequently occurs in tropical and subtropical regions, and it is a major problem to small ruminants [34]. Epidemiologic studies aimed to determine the prevalence of anaplasmosis uses different diagnostic tools, such as microscopic examination of stained blood smears, serological, and molecular tests. The reliability of the diagnostic tests is crucial for accurate diagnosis and estimation of the disease prevalence. Despite microscopic examination and serologic tests are practical and reliable diagnostics to detect *Anaplasma* spp. infection, they have limitations [1, 47]. The accuracy of stained blood smear examination can be hindered by the low number of infected cells, lack of expertise of the examiner, and/or the occurrence of intracellular artifacts [2, 3]. In the early acute phase of infection, serologic assays have limited value, due to the absence of detectable antibodies [5, 42].

Our results showed that the number of infected animals by *Anaplasma* spp. was 43.6% (45.6% in sheep and 40.7% in goats) when examined microscopically compared to 47.4% (49.2% in sheep and 44.7% in goats) using cELISA and 57.4% (59.8% in sheep and 53.7% in goats) by using IFA. On the other hand, the prevalence using PCR was 62.2% (65.1% in sheep and 57.7% in goats), consistent with previous findings [22]. The variability of infection rates determined by different methods maybe attributed to several factors as, age, gender, and species. Previous studies have shown that some cases of anaplasmosis might be missed depending on the detection method used [30, 33].

In this study, sheep had a higher prevalence of anaplasmosis than goats in agreement with previous studies in India and Cyprus [12, 45]. Based on the age factor, it was found that age group I (2 m-2 yrs) had the highest rate of anaplasmosis. Also, gender seemed to play a role whereby anaplasmosis was more prevalent in males than in females. Additionally, *Anaplasma* infection rates were higher among the imported sheep and goats compared with local animals. This could be attributed to the presence of a specific tick vector in the source countries.

*Anaplasma* is routinely diagnosed by microscopic examination of Giemsa stained blood smears and detection of intraerythrocytic *Anaplasma* inclusions. Microscopic examination is suitable for diagnosis of acute anaplasmosis, but it is not applicable for the detection of pre-symptomatic or carrier cases due to low numbers of *Anaplasma* infected cells in circulation, which falls below the detection limit [17, 32].The cELISA currently used for diagnosis of anaplasmosis is based on the use of ANAF16C1 Mab that recognizes the MSP5 antigen in *A. marginale*, *A. central* and *A. ovis*. The MSP-5 antigen is conserved among all known *Anaplasma* spp. [15, 16, 29]. Our results showed that cELISA enabled the detection of more *Anaplasma* cases compared to microscopic examination (62.2% vs. 43.6%, respectively), with 91.9% sensitivity and 86.9% specificity. This was consistent with the findings of Naqid and Zangana [30] who found that the prevalence of *A. ovis* infection in goats was lower (55.9%) using Giemsa stained blood smears compared to cELISA (75.2%).

Indirect fluorescent antibody technique has been recommended and has proven sensitivity for the diagnosis of haemoparasites as *Anaplasma* spp. [35]. Several studies have reported that IFA may be used as an alternative to PCR, CF, and ELISA [10, 23, 38] for the detection of anaplasmosis among sheep and goats [12, 49]. In this study, we found that IFA had high sensitivity (100%) and specificity (91.9%) when compared to the Gold standard (combination of ME and cELISA). A previous study reported a similar level of specificity and sensitivity for IFA when compared with cELISA [22].

Detection of carrier animals is very important, as they play a significant role in the disease epidemiology as reservoirs. Furthermore, it is essential for epidemiologic studies to discriminate between *Anaplasma* species [31]. PCR is reported to be more sensitive than conventional parasitological techniques in different hosts. It also enables the identification of different species [18, 19]. Therefore, we also evaluated PCR for detection of *Anaplasma* species in our animals in comparison with ME, cELISA, and IFA.

The sensitivity of the PCR results was 100% compared to the other diagnostic results. However, in agreement with other studies [30, 32], our results revealed that only 70% of the PCR-positive animals were smear positive. This may be due to animals being examined during the early stage of infection when *Anaplasma*-infected cells are low. Having the ability to detect and differentiate between *Anaplasma* species is important for their accurate diagnosis and better understanding of their burden [11, 42]. Using PCR, we were also able to differentiate between *A. ovis* and *A. phagocytophilum* and to identify animals with mixed infections. In agreement with other studies, our results revealed that 70% of PCR-positive animals were smear positive. This may be due to the animals examined in early stage and there is a low number of *Anaplasma* cells in the circulating blood. Our data and those of others, suggest that PCR may provide a more reliable diagnosis of anaplasmosis particularly during early stages of infection, which would ensure proper management of infected animals and reduction of unnecessary antibiotic use [4, 11].

Comparing the sensitivity of the PCR assay with the cELISA, a lower percentage of animals were seropositive than PCR-positive. A previous study on anaplasmosis seroprevalence using cELISA showed that 88% was seropositive while 76% were positive by using PCR [22]. In our study,47.4% was seropositive while 62.2% were PCR-positive; which means that 14.8% of PCR-positive sera were seronegative, this may occur in the early stage of infection where antibodies have not yet been produced [5].

The prevalence of *A. phagocytophylum* in sheep was 39.2%, and in goats it was 33.3%. While, a lower prevalence of *A. ovis* was reported among sheep and goats, 25.9 and 24.3%, respectively, consistent with previous studies in Cyprus, China, Switzerland, and Italy [12, 37, 44, 49].

The zoonotic potential of *A. phagocytophilum* was previously documented [36, 41].The absence of host specificity of *A. phagocytophilum* allows the infection of the human by animal strains and vice versa [6]. Thus, the high prevalence *A. phagocytophilum* detected in the present study constitutes a risk to humans.

## Conclusions

Proper disease diagnosis requires reliable tests. Therefore, it is important to evaluate the existing diagnostic methods. The evaluation depends on several factors as; whether the test is suitable for the field and/or the laboratory settings; cost; and time required. Microscopic examination provides reliable results, but it is not suitable to diagnose carrier animals. cELISA is known for its ease of use, low cost, and for being quantitative. IFA is an economical and easy method to perform. In the present study, IFA was highly specific and sensitive, but it requires special laboratory settings such as fluorescent microscope. PCR is the most sensitive and reliable diagnostic tool that achieves simultaneous differentiation between different *Anaplasma* subspecies.

## Abbreviations

cELISA: Competitive ELISA
CF: Complement fixation tests
ELISA: Enzyme-linked immunosorbent assay
IFA: Indirect fluorescence antibody
IgG: Immunoglobulin G
Mab: Monoclonal antibody
ME: Microscopic examination
MSP5: A protein epitope of major surface protein 5
OD: The optical density
PBS: Phosphate-buffer saline
PCR: Polymerase chain reaction
